# Exploring Non-Embodied AI-Based Digital Companions for Older Adults in Aging and Care Contexts: Protocol for a Scoping Review

**DOI:** 10.2196/93196

**Published:** 2026-06-24

**Authors:** Lillian Hung, Yi-ting Chiu, Maral Ghodsi

**Affiliations:** 1 IDEA Lab, School of Nursing, University of British Columbia, 5280-5955 University Blvd, Room 4190, Vancouver, BC, V6T 1Z1, Canada, 1 604 208 8317; 2 Department of Kinesiology, Faculty of Health, University of Waterloo, Waterloo, ON, Canada

**Keywords:** non-embodied digital companions, conversational artificial intelligence, conversational agents, chatbots, virtual companions, companionship, social interaction, psychosocial support, older adults, scoping review

## Abstract

**Background:**

Conversational artificial intelligence (AI) technologies are increasingly positioned as a response to social isolation, loneliness, and unmet psychosocial needs across health and care contexts. Non-embodied AI-based digital companions have attracted growing attention for their potential to support companionship, social interaction, communication, and psychosocial well-being among older adults, including people living with dementia. However, the evidence base remains underexplored. Terminology is inconsistently applied, systems are variably defined, and studies are distributed across disciplinary silos, limiting critical investigation of how these technologies are conceptualized, designed, and evaluated.

**Objective:**

This study aims to map and critically synthesize the existing literature on non-embodied AI-based digital companions for older adults in aging, health, and care contexts. The review seeks to describe digital companions, examine methodological approaches, and identify research gaps.

**Methods:**

This protocol follows the Joanna Briggs Institute methodology for scoping reviews and will be reported in accordance with the PRISMA-ScR (Preferred Reporting Items for Systematic Reviews and Meta-Analyses extension for Scoping Reviews guidelines). A comprehensive search will be conducted across multiple electronic databases, including MEDLINE, APA PsycINFO, CINAHL, Scopus, Web of Science, IEEE Xplore, and ACM Digital Library, as well as selected gray literature sources. The search strategy combined terms related to digital companions, conversational AI, aging, and care contexts, including concepts related to companionship, social interaction, communication, loneliness, and psychosocial support.

Eligible studies will include empirical studies involving older adults that examine non-embodied AI-based digital companions designed primarily to support companionship, social interaction, communication, or related psychosocial support in aging, health, and care contexts. Two reviewers (YC and MG) will independently conduct study selection and data charting. Data will be synthesized using descriptive statistics and narrative analysis.

**Results:**

This protocol outlines a systematic approach to identifying, selecting, and synthesizing the existing evidence on non-embodied AI-based digital companions. A preliminary PRISMA (Preferred Reporting Items for Systematic Reviews and Meta-Analyses)-style search flow for the revised database searches identified 2289 records from MEDLINE, CINAHL, and APA PsycINFO. After duplicate removal and eligibility-based removals before screening, 1978 records remained available for title and abstract screening. The full scoping review will summarize study characteristics, populations, contexts, digital companion features, and methodological trends using descriptive tables and narrative synthesis. As of June 2026, the revised database searches had been completed for MEDLINE, CINAHL, and APA PsycINFO, and title and abstract screening was underway. The full scoping review results are expected to be submitted for publication after screening, data charting, and synthesis are completed.

**Conclusions:**

This scoping review is expected to clarify conceptual boundaries, map the scope of current research, and identify knowledge gaps related to non-embodied AI-based digital companions in health, aging, and care contexts. The findings will inform future research, design, and implementation of non-embodied AI-based digital companions.

## Introduction

### Background

The aging population is rapidly increasing globally, with projections indicating that by 2030, one in six individuals will be 60 years and older [1]. Although dementia is not a consequence of aging, it is more common in older age and is a leading cause of disability and frailty [2]. Currently, more than 55 million people worldwide are living with dementia, and it is estimated that by 2050, this figure will reach 139 million [3]. As individuals age and face physical, functional, or cognitive difficulties, the need for ongoing care increases [4]. Long-term care (LTC) and community care settings are the primary environments in which care and support for older adults are received [5].

Social isolation is a global concern that affects a large number of older adults, including people living with dementia, and is highly associated with increased subjective feelings of loneliness [6,7]. Evidence suggests a bidirectional relationship between LTC residency and social isolation, suggesting that being socially isolated can increase the likelihood of institutionalization, and residence in LTC might exacerbate isolation and increase feelings of loneliness [8]. A scoping review by Boamah et al [9] highlighted that increased social isolation among people living in LTC comes from individual factors, such as communication barriers and cognitive impairment, as well as system-level and structural factors, including dependence on care staff and loss of connection with family and friends.

Social isolation and loneliness are distinct yet interrelated concepts. Social isolation refers to an objective, measurable concept characterized by the number of relationships and frequency of social contacts and interactions [10]. Loneliness, on the other hand, is a subjective feeling when the quantity and quality of the social relationships are different from the individual’s desires, needs, and expectations [11]. Both social isolation and loneliness are associated with poor physical and mental health outcomes among older adults, including increased risk of cognitive impairment, cardiovascular and other chronic diseases, as well as anxiety and depression, all of which contribute to reduced quality of life [12].

### Key Concepts and Scope Clarification

With the advent of large language models and conversational artificial intelligence (AI), innovative ways of mitigating social challenges such as isolation and loneliness have emerged [13]. Conversational AI technologies offer natural and simple communication with computers, especially valuable for individuals with low digital literacy or people living with disabilities [14]. AI companion is a broad term that has been used in the literature to describe a range of systems, including robots and virtual conversational agents, that are designed to provide users with emotional support and a sense of bonding, thereby maintaining an ongoing relationship with users [15,16]. Conversational agents are defined as computer-based intelligent systems able to simulate human-like conversations, enabling natural human-computer interaction and expression of empathic responses [17]. Similarly, chatbots are innovative human-like conversation simulators designed to provide companionship, emotional support, and a sense of connection [18].

This scoping review focuses on non-embodied, AI-based digital companions designed for companionship, social connection, and communication support. Some systems may include limited visual elements, such as a static human image or avatar used as a visual anchor. However, these elements do not involve dynamic facial expressions, embodied behaviors, or interactive visual feedback. In this review, such systems are considered non-embodied because the visual component functions primarily as a static interface element rather than as an interactive social embodiment mechanism. In contrast, systems in which embodiment or animated visual representation constitutes a primary design focus or research variable, such as embodied conversational AI systems, are beyond the scope of this review. Other AI technologies, such as AI-driven decision support systems, designed to assist health care professionals in providing more accurate diagnoses, or nonconversational physical robots, are beyond the scope of this review [19].

Non-embodied AI-based digital companions, implemented through conversational AI systems, have been applied in older adults and dementia care for a variety of purposes reported in the literature, including health management, caregiver support, therapeutic interventions, and companionship-related functions [20-29]. For example, prior studies have used these systems to provide medication reminders, deliver health information, support reminiscence activities, and assist caregivers in dementia care contexts [20-22,26-29]. At the same time, a growing body of work has examined their role in promoting social engagement, emotional well-being, and reducing loneliness among older adults [23-25]. However, despite these diverse applications, the focus of this review is specifically on their role in companionship, social interaction, and communication. This focused scope is intentionally adopted to improve conceptual clarity and reduce ambiguity arising from the heterogeneous functions of conversational AI systems. Other functions are considered only when they are integrated into or secondary to a companionship- or communication-oriented system. Despite the promising results mentioned in various applications of conversational AI systems, it is also important to acknowledge their risks, ethical concerns, and implementation considerations. User engagement and acceptability remain important issues. While many older adults often report good engagement and willingness to adopt new technologies, some individuals raise concerns about complicated instructions, lack of knowledge and confidence, health issues like low eyesight, and cost as barriers to using technologies [30].

Several ethical complications have also been raised in the literature. While intended to reduce loneliness, the artificial nature of non-embodied AI-based digital companions may, in some cases, intensify feelings of loneliness and dependency over time. As AI companions become a competitive business, adherence to ethical principles might be overshadowed. Privacy, autonomy, safety, and respect should be carefully considered when developing or implementing AI companions [16]. When it comes to older adults with cognitive challenges, other risks and concerns may arise. For example, the potential inability to distinguish between reality and imagination can lead to confusion and deception [16]. Dehumanization of care is another core concern in older adult care, suggesting that overreliance on AI-based digital companions to provide care-related support could reduce the role of human caregivers to one of oversight rather than relational engagement [31]. Participatory designs tailored to the specific needs of older adults could potentially mitigate or prevent this concern [31].

Aside from ethical considerations, design and implementation strategies are equally important to enhance engagement, acceptability, and efficiency. A qualitative study on tablet-based technologies highlighted the importance of simplified designs, lightweight devices, and labeled buttons [30]. Effective non-embodied AI-based digital companions, like chatbots, should prioritize personalization and adaptability to the user’s interests and cultural background [32]. They should be easy to use and understand, use large fonts and clear language, and have a friendly and engaging interface [18]. Transparency and clear explanation of security and privacy measures are essential to ensure safe and effective implementation of conversational systems among older adults [18]. In this review, we use the term “non-embodied AI-based digital companions” as the primary analytical concept. AI-based refers broadly to systems capable of conversational interaction through rule-based, machine learning, or large language model approaches. Related terms such as chatbots, conversational agents, and virtual companions are treated as examples of, or supporting terminology under, the broader umbrella of non-embodied AI-based digital companions, rather than as separate analytical categories. Terminology use and conceptual scope boundaries are further detailed in Multimedia Appendix 1.

### Rationale

Recent years have seen a growing body of research examining the use of conversational AI systems, including chatbots, virtual agents, and AI companions, to support older adults, people living with dementia, and their caregivers. Existing systematic reviews and scoping reviews have explored related technologies across a range of contexts, such as voice assistants and intelligent virtual agents for older adults, chatbots to support people with dementia and family caregivers, and digital technologies aimed at reducing loneliness and social isolation in aging populations [33-38]. These reviews have contributed important insights into usability, acceptability, and selected outcomes, including social connectedness, mental health, and caregiver support.

However, the existing review literature remains fragmented. Prior reviews focus on narrowly defined technologies, such as voice-enabled assistants or embodied conversational agents, or target specific populations or outcomes, such as dementia-related support or loneliness reduction [35-37]. Terminology and conceptual boundaries vary substantially across studies and reviews, with overlapping use of terms such as conversational agents, chatbots, AI companions, and virtual assistants, often without consistent definitions or scope clarification [33,34,39]. In addition, prior reviews frequently adopt discipline-specific perspectives (eg, health informatics, human-computer interaction, gerontology, or mental health), resulting in a dispersed evidence base that makes it difficult to obtain an integrated overview of how non-embodied AI-based digital companions for companionship and social connection have been conceptualized, implemented, and studied across different care contexts [38-40]. Despite these developments, existing reviews rarely provide a focused synthesis of non-embodied AI-based digital companions specifically in relation to companionship, social interaction, and communication. As a result, the conceptualization and application of these systems for relational and psychosocial purposes remain insufficiently integrated across the literature. Therefore, there is a need for a comprehensive scoping review that systematically maps how non-embodied AI-based digital companions are defined, applied, and studied across populations, settings, and care contexts.

More importantly, while the literature on conversational AI technologies in aging and dementia care is expanding, the evidence base has not yet been comprehensively mapped in a way that captures the breadth of study designs, populations, settings, and application areas relevant to non-embodied AI-based digital companions designed for companionship and social connection. Existing reviews tend to emphasize intervention effectiveness, specific outcomes, or particular technological modalities, rather than systematically describing how these systems are conceptualized, implemented, and studied across diverse care contexts [34,37,38]. As a result, there remains a need to clarify how non-embodied AI-based digital companions are being used, who they are designed for, and in what health and care environments they have been examined.

A scoping review is therefore an appropriate approach to address these needs. Scoping reviews are particularly well-suited to emerging and interdisciplinary fields where concepts, terminology, and study designs are heterogeneous [40]. By systematically identifying and mapping the existing literature, this review aims to provide an overview of how non-embodied AI-based digital companions for companionship and social connection have been defined, applied, and investigated across aging, dementia, and care contexts. This synthesis will help clarify the extent, nature, and characteristics of the current evidence base and inform future research, practice, and policy development in this rapidly evolving area.

#### Objectives

The objective of this scoping review is to map and synthesize the existing literature on non-embodied AI-based digital companions for older adults in health, aging, and care contexts. Specifically, this review aims to describe key concepts, study characteristics, digital companion features, and research trends, and to identify gaps in the current evidence base to inform future research, design, and implementation.

#### Review Questions

##### Primary Review Question

What types of non-embodied AI-based digital companions have been examined in the existing literature for older adults in health, aging, and care contexts?

##### Secondary Review Questions

1. What older adult populations and care-related settings have been studied in relation to non-embodied AI-based digital companions?
2. What study designs and methodological approaches have been used to investigate non-embodied AI-based digital companions for older adults?
3. What key themes, functions, or intended purposes of non-embodied AI-based digital companions are reported, particularly in relation to companionship, social interaction, communication, or related psychosocial support?
4. What gaps in the existing literature can be identified to inform future research, design, and implementation in aging and care contexts?

## Methods

### Study Design

This scoping review protocol is designed in accordance with the Joanna Briggs Institute methodology for scoping reviews [41]. Scoping review methodology was selected because it is well suited to addressing broad and exploratory research questions, particularly in emerging fields where concepts, study designs, and evidence remain heterogeneous and have not yet been comprehensively mapped. The purpose of this review is to systematically identify, examine, and map the existing literature related to non-embodied AI-based digital companions, rather than to assess intervention effectiveness or methodological quality. Therefore, a scoping review approach is more appropriate than a systematic review or meta-analysis. This approach allows for the inclusion of diverse study designs and provides an overview of the extent, nature, and characteristics of the available evidence. The reporting of this scoping review protocol will follow the PRISMA-ScR (Preferred Reporting Items for Systematic Reviews and Meta-Analyses extension for Scoping Reviews) to ensure transparency and methodological rigor [42].

### Eligibility Criteria Population-Concept-Context (PCC)

#### Overview

The eligibility criteria for this scoping review were defined using the population-concept-context (PCC) framework. The PCC framework was selected to ensure conceptual clarity while maintaining alignment with the practical implementation contexts in which digital companion systems are deployed.

#### Population

This review will include studies involving older adults, including older adults with or without cognitive impairment. Studies involving caregivers, family members, or care staff will be eligible only when the study reports interaction with, use of, or perceptions of the digital companion in relation to older adult users. Studies focused exclusively on children, adolescents, younger adults, or staff workflows without older adult user-related digital companion interaction will be excluded.

#### Concept

The concept of interest is the use and impact of non-embodied AI-based digital companions, including conversational agents, virtual companions, and chatbot-based systems designed primarily to support companionship, social interaction, communication, or related psychosocial support. Studies focusing solely on unrelated digital technologies, such as general telehealth platforms without companion-like interaction, monitoring systems without interactive components, or AI-driven clinical decision-support tools, will be excluded. Studies involving physical robots or robotic companions will also be excluded. Studies in which embodied visual representation, such as animated avatars, facial expressions, or embodied conversational agents, constitutes a primary intervention component or analytical focus will be excluded.

#### Context

This review will consider studies conducted in any setting, including but not limited to community settings, private homes, LTC facilities, assisted living environments, health care institutions, and virtual or remote contexts. No geographic restrictions will be applied.

#### Types of Sources

This scoping review will include empirical studies of all designs, including qualitative, quantitative, and mixed-methods research, as well as pilot studies and feasibility studies. Review articles will be excluded but will be screened for relevant references. Conference abstracts will be included only if sufficient methodological and outcome information is available. Only studies published in English will be considered. Studies published from 2000 onward will be included to capture the conceptual and technological development of digital companion systems and related conversational AI technologies. Earlier literature predates the technical capabilities required for interactive, dialogue-based systems and is therefore unlikely to be relevant to the focus of this review. A detailed overview of the eligibility criteria based on the PCC framework is provided in Multimedia Appendix 2.

### Information Sources

This scoping review will identify relevant literature through a comprehensive search of electronic bibliographic databases, gray literature sources, and preprint servers. The selection of information sources was designed to ensure broad coverage across health, aging, nursing, psychology, and technology-related disciplines relevant to non-embodied AI-based digital companions.

The following electronic databases will be searched: MEDLINE (via Ovid), APA PsycINFO, CINAHL (via EBSCOhost), Scopus, Web of Science Core Collection, IEEE Xplore, and ACM Digital Library. Multiple interdisciplinary databases were included to maximize coverage across health sciences, psychology, gerontology, and human-computer interaction literature. To supplement peer-reviewed literature and capture relevant gray literature, additional searches will be conducted in Google Scholar. Gray literature sources such as reports, conference materials, and theses will be considered when they meet the eligibility criteria. In Google Scholar, keyword-based searches will be performed, and the first 200 results sorted by relevance will be screened for each search. In addition, to identify emerging and unpublished research, preprint servers, including medRxiv and arXiv, will also be searched using keyword-based strategies aligned with the core search concepts. Preprints will be clearly labeled during data charting and synthesis to distinguish them from peer-reviewed publications.

All searches will be documented in detail, including the database name, search date, search strategy, and number of records retrieved. As part of the revised protocol documentation, a preliminary PRISMA-style search flow is provided for the revised searches conducted in three core health and psychology databases: MEDLINE, APA PsycINFO, and CINAHL. The full search strategies will be provided in a Multimedia Appendix 3.

### Search Strategy

The search strategy was developed through an initial exploratory search conducted in Ovid MEDLINE to identify relevant articles and terminology. Following refinement of the conceptual scope, the final search strategy combined terms related to non-embodied conversational AI and digital companions with adult, aging, and care-context terms. To improve specificity, core terms such as chatbot, conversational agent, AI companion, digital companion, and virtual companion were searched directly, while broader terms such as virtual assistant, voice assistant, avatar, and human-computer interaction were combined with functional-scope terms related to companionship, social interaction, communication, loneliness, social isolation, emotional support, or psychosocial support.

Although the review eligibility criteria focus on older adults and aging- or care-related contexts, the search strategy retained broader adult and care-context terms at the database search stage to maximize sensitivity and avoid missing studies in which older adult populations were not explicitly indexed or described in titles and abstracts. Older-adult relevance will be assessed during title and abstract screening, full-text screening, and data charting.

The full revised search strategies for MEDLINE, APA PsycINFO, and CINAHL are provided in Multimedia Appendix 3. The MEDLINE strategy was first developed in Ovid and then adapted for other databases according to database-specific indexing terms, search syntax, and search functionalities. Search strategies for additional databases will be adapted using the same conceptual logic and documented in the full review. The search will aim to identify both published and unpublished literature. In addition to database searches, the reference lists of studies included after full-text screening will be examined to identify additional relevant sources.

### Study Selection

All records identified through the database searches will be imported into a reference management software, where duplicate records will be identified and removed. Following de-duplication, the study selection process will be conducted in two sequential stages: (1) title and abstract screening and (2) full-text screening. In the first stage, 2 reviewers (YC and MG) will independently screen the titles and abstracts of all retrieved records against the predefined eligibility criteria. Studies that do not meet the inclusion criteria will be excluded at this stage. Records that appear potentially relevant, or for which eligibility is unclear, will be retained for full-text review.

In the second stage, the full texts of all potentially eligible studies will be retrieved and independently assessed by 2 reviewers (YC and MG) using the same eligibility criteria. Reasons for exclusion at the full-text stage will be documented and reported in the final scoping review. Any disagreements between reviewers at either screening stage will be resolved through discussion. If consensus cannot be reached, a third reviewer (YK) will be consulted to make the final decision. The study selection process will be documented and presented using a PRISMA-ScR flow diagram. For this protocol revision, a preliminary PRISMA-style search flow is provided to document the revised database searches, duplicate removal, and records available for title and abstract screening. Full title and abstract screening, full-text screening, and final study inclusion will be completed in the full review.

### Data Charting

Data charting will be conducted to systematically extract and organize key information from the included studies in alignment with the review objectives and PCC framework. A standardized data charting form will be developed by the research team to ensure consistency and transparency throughout the extraction process. The draft charting form will capture core bibliographic information (eg, authors, year of publication, and country), study characteristics (eg, study design and setting), population characteristics (eg, age group and user type), and key features of the digital companion systems examined (eg, type of technology, interaction modality, and functional focus). Additional data items relevant to the review questions, such as study aims, reported outcomes, and key findings, will also be extracted.

Prior to full data charting, the draft charting form will be pilot-tested on a small subset of included studies to assess clarity, completeness, and consistency. The form may be refined iteratively based on team discussions during this pilot phase. Any modifications to the charting form will be documented and reported in the final scoping review.

Data charting will be performed independently by 2 reviewers (YC and MG). Any discrepancies in extracted data will be discussed and resolved through consensus. If necessary, a third reviewer (YK) will be consulted. As this scoping review aims to map the extent, nature, and characteristics of the existing literature, no formal assessment of methodological quality or risk of bias will be conducted.

### Data Synthesis and Presentation

The extracted data will be synthesized using descriptive and narrative approaches consistent with the objectives of a scoping review. Findings will be organized and mapped to provide an overview of the extent, nature, and characteristics of the existing literature on non-embodied AI-based digital companions. A summary table will be used to present key study characteristics and extracted data, including study design, population, context, and features of the digital companion systems. The tabulated results will be accompanied by a narrative synthesis that summarizes patterns, trends, and gaps in the literature in relation to the review questions.

The narrative synthesis will group findings into thematic categories derived inductively from the charted data. These themes may include, but are not limited to, types of digital companions, interaction modalities, implementation contexts, and reported functions or impacts. Both qualitative and quantitative evidence will be summarized descriptively to reflect the breadth of the literature. The results will be reported in accordance with the PRISMA-ScR, including a flow diagram to illustrate the study selection process.

### Ethical Considerations

Ethics approval is not required for this scoping review, as it does not involve the collection or analysis of primary data from human participants and is based solely on publicly available literature. Furthermore, findings from this scoping review will be disseminated through peer-reviewed publications and academic conferences. The results will be of interest to researchers in gerontology, nursing, digital health, and human-computer interaction, as well as to health and care practitioners and technology designers involved in the development or implementation of AI-based digital companions. The findings may also inform policymakers and system-level stakeholders interested in aging and digital health.

## Results

The results of this scoping review will be reported in accordance with the PRISMA-ScR. The study selection process will be summarized using a PRISMA flow diagram in the full review, detailing the number of records identified, screened, assessed for eligibility, and included, along with reasons for exclusion at the full-text screening stage.

For this protocol revision, a preliminary PRISMA-style search flow for the revised database searches is provided in Multimedia Appendix 4. This preliminary flow document records identified from three core databases, duplicate removal, eligibility-based removals before screening, and records available for title and abstract screening. The revised searches identified 2289 records across MEDLINE (n=1379), CINAHL (n=597), and APA PsycINFO (n=313). After removing 306 duplicate records and 5 records for eligibility-based reasons before screening, including 4 records published before 2000 and 1 non-English record, 1978 records remained available for title and abstract screening. The characteristics of the included studies will be presented in tabular form in the full review, summarizing key information such as authors, year of publication, country, study design, setting, population characteristics, and the type of non-embodied AI-based digital companion examined. This descriptive mapping will provide an overview of the scope, distribution, and methodological features of the existing literature.

The findings will be synthesized and organized thematically to address the review questions. Results will be presented through a combination of tables and narrative summaries, focusing on: (1) the types and characteristics of non-embodied AI-based digital companions studied; (2) the populations and care contexts in which these systems have been examined; (3) the study designs and methodological approaches used; and (4) the key functions, themes, or intended purposes reported in relation to digital companion use. Identified research gaps, trends, and areas requiring further investigation will be highlighted to inform future research and development in this emerging field. As this is a scoping review, no formal assessment of methodological quality or risk of bias will be conducted, and the results will be interpreted descriptively to provide a comprehensive map of the available evidence. As of June 2026, the revised database searches had been completed for MEDLINE, CINAHL, and APA PsycINFO, and title and abstract screening was underway. The full scoping review results are expected to be submitted for publication after screening, data charting, and synthesis are completed.

## Discussion

### Anticipated Principal Findings

This scoping review is expected to provide a structured map of how non-embodied AI-based digital companions for older adults have been conceptualized, designed, implemented, and evaluated across health, aging, and care contexts. We anticipate that the review will show that the literature remains conceptually fragmented, with overlapping use of terms such as chatbots, conversational agents, virtual companions, voice assistants, and AI companions. By applying a clearer conceptual boundary focused on companionship, social interaction, communication, and related psychosocial support, this review will clarify what types of systems have been studied and how they have been positioned in relation to social connection and care.

We also anticipate that the evidence base will be heterogeneous in terms of study design, target populations, settings, and reported functions. While some studies may focus directly on companionship, loneliness, or social support, others may examine related functions such as communication support, engagement, reminiscence, or caregiver-facing support when these are integrated into companionship- or communication-oriented systems. The review is therefore expected to identify both the breadth of existing research and the limits of current evidence, particularly regarding how these systems are evaluated and how outcomes are conceptualized for older adults across aging and care-related contexts.

### Comparison With Prior Work

Existing reviews have examined related technologies, including voice assistants, chatbots for dementia care, digital interventions for loneliness and social isolation, and embodied conversational agents. However, prior reviews often focus on a specific technology type, population, or outcome. As a result, the broader conceptual landscape of non-embodied AI-based digital companions remains difficult to interpret across disciplinary boundaries.

This review differs from prior work by focusing specifically on non-embodied AI-based digital companions as a conceptual category, while distinguishing these systems from physical robots, monitoring-only technologies, and clinical decision-support tools. Rather than evaluating intervention effectiveness alone, this review will map how these systems are defined, what functions they are designed to support, what populations and settings have been studied, and where conceptual or methodological gaps remain. This approach is particularly relevant for an emerging field in which terminology and system boundaries remain inconsistent.

### Strengths and Limitations

A key strength of this scoping review is its conceptually focused approach. By refining the scope to non-embodied AI-based digital companions designed primarily for companionship, social interaction, communication, or related psychosocial support, the review aims to reduce ambiguity while still capturing interdisciplinary evidence across health, aging, nursing, psychology, and human-computer interaction. The use of the PCC framework and PRISMA-ScR guidance will support methodological transparency and systematic reporting. Another strength is the use of a revised search strategy designed to balance sensitivity and specificity. The search strategy includes core terms such as chatbot, conversational agent, AI companion, digital companion, and virtual companion, while broader terms such as virtual assistant, avatar, and human-computer interaction are combined with functional-scope terms related to companionship, social interaction, communication, loneliness, social isolation, emotional support, or psychosocial support.

Several limitations should also be acknowledged. First, because this is a scoping review, the study will not assess the methodological quality or risk of bias of included studies and will not draw conclusions about intervention effectiveness. Second, limiting inclusion to English-language publications from 2000 onward may exclude some relevant studies. Third, given the rapid development of conversational AI technologies, new systems and studies may emerge after the search is completed. Finally, because terminology in this field is inconsistent, some relevant studies may use terms that differ from those included in the search strategy, although reference list screening and supplementary searches will help mitigate this limitation.

### Future Directions

The findings of this review are expected to inform future research by identifying gaps in study design, population coverage, implementation context, and outcome conceptualization. In particular, the review may highlight the need for clearer terminology, more consistent reporting of system features, and stronger attention to ethical and implementation considerations, especially in aging and care settings. Future research should examine not only whether digital companions reduce loneliness or improve social connection, but also how users understand, engage with, and integrate these systems into daily life. Future research should also examine how older adults with different cognitive, social, cultural, and care-related needs use and experience these technologies, and whether design priorities should vary across community, assisted living, LTC, and dementia care contexts.

### Dissemination Plan

Findings from this scoping review will be disseminated through peer-reviewed publications and academic conference presentations. The results may also be shared with researchers, practitioners, care organizations, and technology developers interested in the design and implementation of non-embodied AI-based digital companions. By clarifying conceptual boundaries and mapping the current evidence base, this review is intended to support more informed research, design, and implementation decisions in the rapidly evolving field of conversational AI and digital companion technologies.

## Supplementary material

10.2196/93196 Multimedia Appendix 1 Terminology use and conceptual scope clarification.

10.2196/93196 Multimedia Appendix 2 Eligibility criteria based on the population–concept–context (PCC) framework.

10.2196/93196 Multimedia Appendix 3 Revised search strategies for core databases.

10.2196/93196 Multimedia Appendix 4 Preliminary PRISMA-style search flow for revised database searches.

10.2196/93196 Checklist 1 PRISMA-ScR checklist.

10.2196/93196 Peer Review Report 1 Peer review report from Mitacs Elevate Postdoctoral Fellowship Program, Health Research BC–Mitacs Industry-Based partnership (Canada).

## Abbreviations

AI: artificial intelligence
LTC: long-term care
PCC: population-concept-context
PRISMA-ScR: Preferred Reporting Items for Systematic Reviews and Meta-Analyses extension for Scoping Reviews

## References

[1] (2022). Ageing and health. World Health Organization.

[2] LoGiudice D, Watson R (2014). Dementia in older people: an update. Intern Med J.

[3] Dementia facts & figures. Alzheimer’s Disease International.

[4] Vinsnes AG, Nakrem S, Harkless GE, Seim A (2012). Quality of care in Norwegian nursing homes - typology of family perceptions. J Clin Nurs.

[5] Sehatkarlangrodi S, Zargoush M, Longo CJ, Ghazalbash S (2025). Shaping older adults’ care policy: a scoping review of key determinants in post-acute and community reintegration transitions. BMC Health Serv Res.

[6] Cudjoe TKM, Roth DL, Szanton SL, Wolff JL, Boyd CM, Thorpe RJ (2020). The epidemiology of social isolation: national health and aging trends study. J Gerontol B Psychol Sci Soc Sci.

[7] Hajek A, König HH (2025). Prevalence of loneliness and social isolation among individuals with mild cognitive impairment or dementia: systematic review and meta-analysis. BJPsych Open.

[8] Brock AM, O’Sullivan P (1985). A study to determine what variables predict institutionalization of elderly people. J Adv Nurs.

[9] Boamah SA, Weldrick R, Lee TSJ, Taylor N (2021). Social isolation among older adults in long-term care: a scoping review. J Aging Health.

[10] Gierveld JDJ, Tilburg TV (2006). A 6-Item scale for overall, emotional, and social loneliness: confirmatory tests on survey data. Res Aging.

[11] Peplau LA, Perlman D (1982). Loneliness: A Sourcebook of Current Theory, Research and Therapy.

[12] Puyané M, Chabrera C, Camón E, Cabrera E (2025). Uncovering the impact of loneliness in ageing populations: a comprehensive scoping review. BMC Geriatr.

[13] Hollanek T, Sobey A (2025). AI companions for health and mental wellbeing: opportunities, risks and policy implications.

[14] Pradhan A, Mehta K, Findlater L (2018). "Accessibility came by accident”: use of voice-controlled intelligent personal assistants by people with disabilities.

[15] Lim MY, Zacarias M, Oliveira JV (2012). Human-Computer Interaction: The Agency Perspective.

[16] Ciriello R, Hannon O, Chen AY, Vaast E (2024). Ethical tensions in human-AI companionship: a dialectical inquiry into replika.

[17] Tsiourti C, Quintas J, Ben-Moussa M, Hanke S, Nijdam NA, Konstantas D, Bi Y, Kapoor S, Bhatia R (2018). Intelligent Systems and Applications.

[18] Rodríguez-Martínez A, Amezcua-Aguilar T, Cortés-Moreno J, Jiménez-Delgado JJ (2024). Qualitative analysis of conversational chatbots to alleviate loneliness in older adults as a strategy for emotional health. Healthcare (Basel).

[19] Elhaddad M, Hamam S (2024). AI-driven clinical decision support systems: an ongoing pursuit of potential. Cureus.

[20] Shade MY, Yan C, Jones VK, Boron JB (2025). Interactive AI routines for pain symptoms and loneliness in older adults. Pain Manag Nurs.

[21] Blocker KA, Kadylak T, Koon LM, Kovac CE, Rogers WA (2020). Digital home assistants and aging: initial perspectives from novice older adult users. Proc Hum Factors Ergon Soc Annu Meet.

[22] Balasubramanian GV, Beaney P, Chambers R (2021). Digital personal assistants are smart ways for assistive technology to aid the health and wellbeing of patients and carers. BMC Geriatr.

[23] Yang Y, Wang C, Xiang X, An R (2025). AI applications to reduce loneliness among older adults: a systematic review of effectiveness and technologies. Healthcare (Basel).

[24] Demiris G, Thompson HJ, Lazar A, Lin SY (2016). Evaluation of a digital companion for older adults with mild cognitive impairment. AMIA Annu Symp Proc.

[25] Jones VK, Yan C, Shade MY (2024). Reducing loneliness and improving social support among older adults through different modalities of personal voice assistants. Geriatrics (Basel).

[26] Sun J, Zhang Z, Wang M (2025). Chorus of the past: toward designing a multi-agent conversational reminiscence system with digital artifacts for older adults.

[27] Tam W, Poon SN, Mahendran R, Kua EH, Wu XV (2021). The effectiveness of reminiscence-based intervention on improving psychological well-being in cognitively intact older adults: a systematic review and meta-analysis. Int J Nurs Stud.

[28] Boiting M, Tschorn N, Suravee S, Cunha A, Paiva A, Pereira S (2024). Wireless Mobile Communication and Healthcare.

[29] Cheng ST, Ng PHF (2025). The PDC30 chatbot—development of a psychoeducational resource on dementia caregiving among family caregivers: mixed methods acceptability study. JMIR Aging.

[30] Vaportzis E, Clausen MG, Gow AJ (2017). Older adults perceptions of technology and barriers to interacting with tablet computers: a focus group study. Front Psychol.

[31] Rubeis G (2020). The disruptive power of Artificial Intelligence. Ethical aspects of gerontechnology in elderly care. Arch Gerontol Geriatr.

[32] da Paixao Pinto N, dos Santos Franca JB, de Sa Sousa HP, Vivacqua AS, Garcia ACB (2021). Conversational agents for elderly interaction.

[33] Ruggiano N, Brown EL, Roberts L (2021). Chatbots to support people with dementia and their caregivers: systematic review of functions and quality. J Med Internet Res.

[34] Boumans R, van de Sande Y, Thill S, Bosse T (2022). Voice-enabled intelligent virtual agents for people with amnesia: systematic review. JMIR Aging.

[35] Castro Martínez E, Hernández Encuentra E, Pousada Fernández M (2025). Voice assistants’ influence on loneliness in older adults: a systematic review. Disabil Rehabil Assist Technol.

[36] Rai HK, Kernaghan D, Schoonmade L, Egan KJ, Pot AM (2022). Digital technologies to prevent social isolation and loneliness in dementia: a systematic review. J Alzheimers Dis.

[37] Huang Y, Zhou Q, Piper AM (2025). Designing conversational AI for aging: a systematic review of older adults’ perceptions and needs.

[38] Welch V, Ghogomu ET, Barbeau VI (2023). Digital interventions to reduce social isolation and loneliness in older adults: an evidence and gap map. Campbell Syst Rev.

[39] Kiuchi K, Otsu K, Hayashi Y (2024). Psychological insights into the research and practice of embodied conversational agents, chatbots and social assistive robots: a systematic meta-review. Behav Inf Technol.

[40] Huq SM, Maskeliūnas R, Damaševičius R (2024). Dialogue agents for artificial intelligence-based conversational systems for cognitively disabled: a systematic review. Disabil Rehabil Assist Technol.

[41] Peters M, Godfrey C, McInerney P, Munn Z, Tricco A, Khalil H, Aromataris E, Lockwood C, Porritt K, Pilla B, Jordan Z (2024). JBI Manual for Evidence Synthesis JBI.

[42] Tricco AC, Lillie E, Zarin W (2018). PRISMA Extension for Scoping Reviews (PRISMA-ScR): checklist and explanation. Ann Intern Med.

