# Supplementary material for: Exploring Non-Embodied AI-Based Digital Companions for Older Adults in Aging and Care Contexts: Protocol for a Scoping Review
Source: JMIR Res Protoc. 2026 Jun 24;15:e93196. doi: 10.2196/93196 (PMC13294803; doi:10.2196/93196)
Supplement: Multimedia Appendix 1 [file resprot-v15-e93196-s001.docx]

# Multimedia Appendix 4: Terminology Use and Conceptual Scope Clarification

## Purpose

This appendix describes the terminology framework used in this scoping review to ensure conceptual clarity and consistent application across sections. Given the heterogeneous and overlapping use of terms in the literature (e.g., conversational AI, chatbots, virtual agents, AI companions), explicit terminology alignment was applied throughout study selection, data charting, and synthesis.

## Core Analytical Term

### Primary Term

**Non-embodied AI-based digital companions** refer to software-based conversational systems designed primarily to provide companionship, social interaction, communication, or related psychosocial support through interactive dialogue, without a physical robotic body. In this review, “AI-based” is used broadly to refer to systems capable of conversational interaction through rule-based, machine learning, or large language model approaches. Some systems may include limited visual elements, such as a static human image or avatar used as a visual anchor. However, these elements do not involve dynamic facial expressions, embodied behaviors, or interactive visual feedback. In this review, such systems are considered non-embodied because the visual component functions primarily as a static interface element rather than as an interactive social embodiment mechanism. This term is used consistently throughout the manuscript to denote the primary analytical unit of the review.

### Supporting Terminology

**Conversational AI systems** are used descriptively to refer to the technological mechanisms through which non-embodied AI-based digital companions are implemented (e.g., natural language processing, dialogue management). It is not used as the primary analytical subject of the review.

**Conversational AI technologies** are used selectively in background sections to describe broad technological developments or trends, and not to define the scope, objectives, or eligibility criteria of the review.

**AI companions** are acknowledged as a broad term used in the literature to describe diverse systems, including physical robots and virtual agents. In this review, the term is referenced only when describing prior literature and not as a substitute for the primary analytical term.

### (3) Functional Scope

In this review, non-embodied AI-based digital companions are conceptualized as systems primarily designed to support companionship, social interaction, communication, or related psychosocial support through conversational engagement. Other functions, such as health information, medication reminders, pain routines, reminiscence activities, or caregiver support, are considered only when they are integrated into or secondary to a companionship-, communication-, or psychosocial-support-oriented system.

## Scope Boundaries and Exclusions

The following systems are considered outside the scope of this review:

- Physical or robotic systems with embodied interaction (e.g., social robots, robotic pets).
- Systems in which embodiment or animated visual representation (e.g., facial expressions, gestures, animated avatars) constitutes a primary intervention component or analytical focus.
- AI systems designed solely for diagnostic, administrative, or decision-support purposes without a conversational or companionship function.
- General telehealth platforms, monitoring systems, or assistive technologies without companion-like conversational interaction.

## Application Across Review Stages

This terminology framework was applied consistently across the formulation of objectives and review questions, eligibility criteria (PCC framework), data charting, synthesis, and reporting of findings, supporting transparency and conceptual coherence.
