# Supplementary material for: Exploring Non-Embodied AI-Based Digital Companions for Older Adults in Aging and Care Contexts: Protocol for a Scoping Review
Source: JMIR Res Protoc. 2026 Jun 24;15:e93196. doi: 10.2196/93196 (PMC13294803; doi:10.2196/93196)
Supplement: Multimedia Appendix 2 [file resprot-v15-e93196-s002.docx]

## **Multimedia Appendix 1**. Eligibility criteria based on the Population–Concept–Context (PCC) framework

| **Component** | **Inclusion Criteria (Include)** | **Exclusion Criteria (Exclude)** |
| --- | --- | --- |
| **Population** | • Older adults, including older adults with or without cognitive impairment  • Studies involving caregivers, family members, or care staff may be included when the study reports interaction with, use of, or perceptions of the digital companion in relation to older adult users | • Children, adolescents, or younger adult-only populations  • Studies strictly focused on staff workflows, clinical operations, or caregiver burden without older adult user-related digital companion interaction |
| **Concept** | • Non-embodied AI-based digital companions, including conversational agents, virtual companions, and chatbot-based systems  • Software-based conversational systems designed primarily to support companionship, social interaction, communication, or related psychosocial support  • Systems that enable two-way conversational or interactive engagement through text, voice, or other non-robotic conversational modalities  • Systems with limited static, non-interactive visual representations, such as a static human image or avatar used as a visual anchor, may be included if these elements do not involve dynamic facial expressions, embodied behaviors, or interactive visual feedback and are not a primary intervention component or analytical focus.• Other functions, such as health information, medication reminders, reminiscence activities, or caregiver support, may be included only when integrated into or secondary to a companionship-, communication-, or psychosocial-support-oriented system | • Physical robots or embodied robotic systems, such as social robots or robotic pets  • Embodied conversational agents or systems in which animated visual representation, facial expressions, gestures, or embodiment constitute a primary design feature or analytical focus  • General telehealth platforms, monitoring systems, or assistive technologies without companion-like conversational interaction  • AI-driven clinical decision-support, diagnostic, administrative, or algorithmic tools without a companionship, communication, or psychosocial-support function  • Systems focused solely on reminders, health information, care coordination, or task management without companion-like conversational engagement |
| **Context** | • All settings, including home, community, long-term care, assisted living, healthcare, and virtual or remote environments  • No geographic restrictions | • None, provided the population and concept criteria are met |
