# Supplementary material for: Exploring Non-Embodied AI-Based Digital Companions for Older Adults in Aging and Care Contexts: Protocol for a Scoping Review
Source: JMIR Res Protoc. 2026 Jun 24;15:e93196. doi: 10.2196/93196 (PMC13294803; doi:10.2196/93196)
Supplement: Multimedia Appendix 3 [file resprot-v15-e93196-s003.docx]

## **Multimedia Appendix 2**: Revised Search Strategies for Core Databases

**Overview of Revised Search Logic**

The revised search strategy was developed to better align with the updated conceptual scope of this scoping review. Multiple interdisciplinary databases were included to maximize coverage across health sciences, psychology, gerontology, and human-computer interaction literature.

The final search logic was:

[B1 OR (B2 AND B3)] AND C

Where:

B1 = Core digital companion / conversational AI terms
B2 = Broader virtual assistant / avatar / human-computer interaction terms
B3 = Companionship / social interaction / communication functional-scope terms
C = Adult / aging / care-context terms

This approach allowed core digital companion and conversational AI terms to be searched directly, while broader terms such as virtual assistant, avatar, and human-computer interaction were retained only when combined with functional-scope terms related to companionship, social interaction, communication, loneliness, social isolation, emotional support, or psychosocial support.

**A1. MEDLINE via Ovid**

**Database:** MEDLINE
**Interface:** Ovid
**Search date:** May 3, 2026
**Final line:** #9
**Records retrieved:** 1,379

1. ("AI companion*" OR "digital companion*" OR "virtual companion*" OR chatbot* OR "conversational agent*").tw,kf.
2. ("virtual assistant*" OR "smart assistant*" OR "virtual agent*" OR "voice assistant*" OR "intelligent virtual agent*" OR avatar* OR "human-computer interaction").tw,kf.
3. (companion* OR companionship OR "social interaction" OR "social connection" OR communication OR loneliness OR "social isolation" OR "emotional support" OR "psychosocial support").tw,kf.
4. 1 OR (2 AND 3)
5. (adult* OR "older adult*" OR senior* OR elderly OR "long-term care" OR LTC OR "nursing home*" OR "assisted living").tw,kf.
6. exp Aged/ OR Adult/ OR Middle Aged/ OR Young Adult/ OR Homes for the Aged/ OR exp Long-Term Care/ OR exp Nursing Homes/
7. 5 OR 6
8. 4 AND 7

9. limit 8 to (English language and yr="2000 -Current")

**A2. CINAHL via EBSCOhost**

**Database:** CINAHL
**Interface:** EBSCOhost
**Search date:** May 3, 2026
**Final line:** S8
**Records retrieved:** 597

S1: TX("AI companion*" OR "digital companion*" OR "virtual companion*" OR chatbot* OR "conversational agent*")
S2: TX("virtual assistant*" OR "smart assistant*" OR "virtual agent*" OR "voice assistant*" OR "intelligent virtual agent*" OR avatar* OR "human-computer interaction")
S3: TX(companion* OR companionship OR "social interaction" OR "social connection" OR communication OR loneliness OR "social isolation" OR "emotional support" OR "psychosocial support")
S4: S1 OR (S2 AND S3)
S5: (MH "Aged+" OR MH "Adults+" OR MH "Residential Care+")
S6: TX(adult* OR "older adult*" OR senior* OR elderly OR "long-term care" OR LTC OR "nursing home*" OR "assisted living")
S7: S5 OR S6
S8: S4 AND S7

**A3. APA PsycINFO via EBSCOhost**

**Database:** APA PsycINFO
**Interface:** EBSCOhost
**Search date:** May 3, 2026
**Final line:** S8
**Records retrieved:** 313

S1: TX("AI companion*" OR "digital companion*" OR "virtual companion*" OR chatbot* OR "conversational agent*")

S2: TX("virtual assistant*" OR "smart assistant*" OR "virtual agent*" OR "voice assistant*" OR "intelligent virtual agent*" OR avatar* OR "human-computer interaction")

S3: TX(companion* OR companionship OR "social interaction" OR "social connection" OR communication OR loneliness OR "social isolation" OR "emotional support" OR "psychosocial support")

S4: S1 OR (S2 AND S3)

S7: TX("older adult*" OR senior* OR elderly OR "long-term care" OR LTC OR "nursing home*" OR "assisted living")

S8: S4 AND S7

Note. Subject heading searches for adult/aging/residential care terms in APA PsycINFO via EBSCOhost did not function consistently in the available interface. Therefore, the final APA PsycINFO strategy used text-word context terms for older adults and care-related settings.

| **Database** | **Interface** | **Final line** | **Records retrieved** |
| --- | --- | --- | --- |
| MEDLINE | Ovid | #49 | 1,379 |
| CINAHL | EBSCOhost | S8 | 597 |
| APA PsycINFO | EBSCOhost | S8 | 313 |
| Total records identified |  |  | 2,289 |

After deduplication, 306 duplicate records were removed. An additional 5 records were removed before screening because they were published before 2000 (n = 4) or were non-English records (n = 1). A total of 1,978 records remained available for title and abstract screening.
