# Supplementary figures and images for: Exploring Non-Embodied AI-Based Digital Companions for Older Adults in Aging and Care Contexts: Protocol for a Scoping Review
Source: JMIR Res Protoc. 2026 Jun 24;15:e93196. doi: 10.2196/93196 (PMC13294803; doi:10.2196/93196)

# Multimedia Appendix 5: Preliminary PRISMA-Style Search Flow for Revised Database Searches


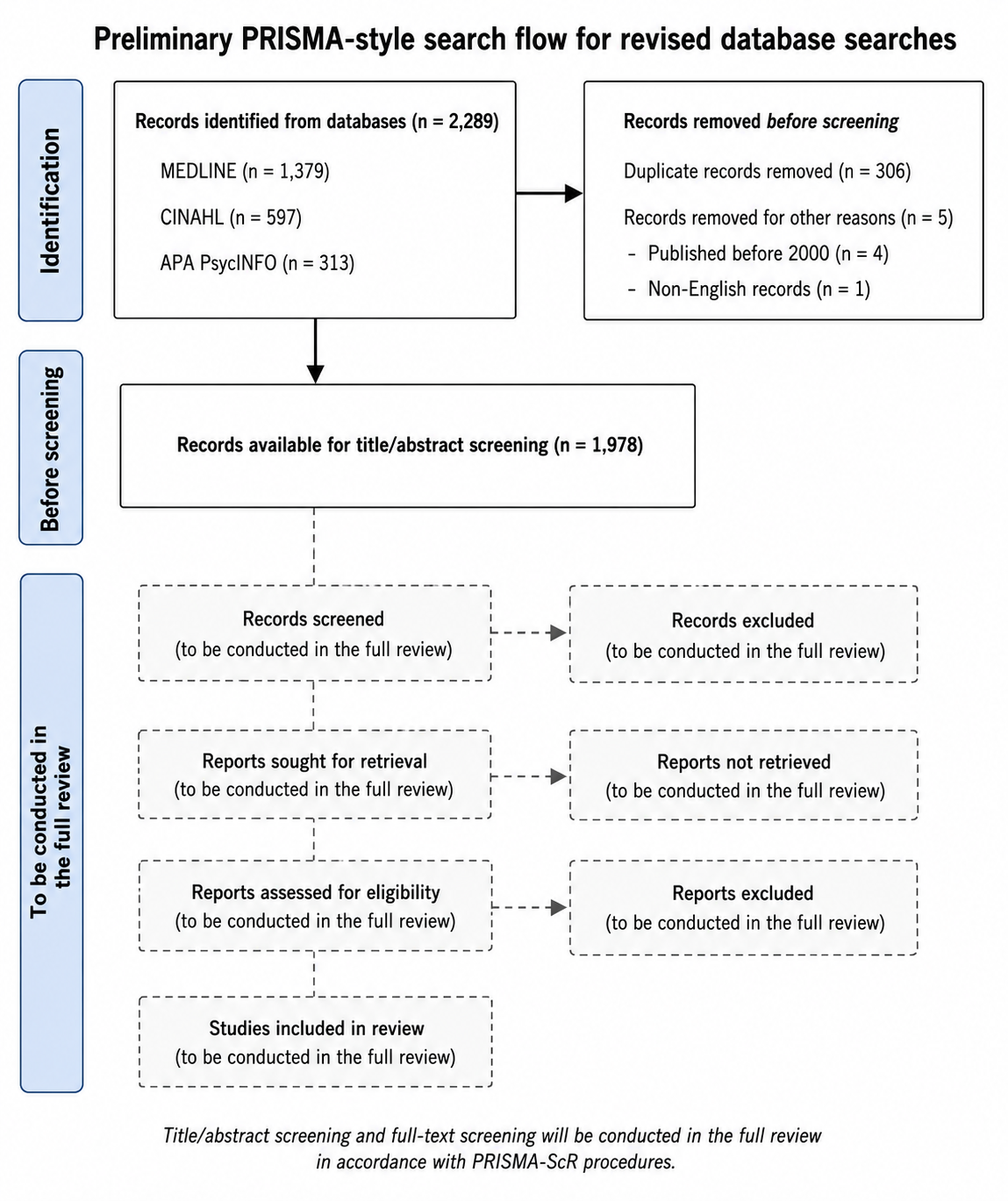

Supplement: Multimedia Appendix 4 [file resprot-v15-e93196-s004.docx]
