# Supplementary material for: Exploring Non-Embodied AI-Based Digital Companions for Older Adults in Aging and Care Contexts: Protocol for a Scoping Review
Source: JMIR Res Protoc. 2026 Jun 24;15:e93196. doi: 10.2196/93196 (PMC13294803; doi:10.2196/93196)
Supplement: Checklist 1 [file resprot-v15-e93196-s005.docx]

## **Multimedia Appendix 3: PRISMA-ScR Checklist**

| Section | Item | PRISMA-ScR Requirement | Reported? | Location |
| --- | --- | --- | --- | --- |
| TITLE | 1 | Identify the report as a scoping review protocol | Yes | Title page |
| ABSTRACT | 2 | Provide structured summary | Yes | Abstract |
| INTRODUCTION | 3 | Rationale | Yes | Introduction |
|  | 4 | Objectives | Yes | Objectives |
| METHODS | 5 | Protocol/registration | Not registered; this scoping review protocol was not registered in PROSPERO as scoping reviews are not currently eligible for registration. | — |
|  | 6 | Eligibility criteria (PCC) | Yes | Methods – Eligibility |
|  | 7 | Information sources | Yes | Methods – Information Sources; Multimedia Appendix 2 |
|  | 8 | Planned search strategy | Yes | Methods – Search Strategy; Multimedia Appendix 2 (revised search strategies for MEDLINE, CINAHL, and APA PsycINFO) |
|  | 9 | Selection process | Yes | Methods – Study Selection |
|  | 10 | Data charting process | Yes | Methods – Data Charting |
|  | 11 | Data items | Yes | Methods – Data Charting |
|  | 12 | Critical appraisal (optional) | Not planned | — |
|  | 13 | Synthesis plan | Yes | Methods – Data Synthesis |
| RESULTS | 14–18 | Preliminary search flow reported for revised database searches; full study selection results not applicable for protocol | Partially reported; preliminary search flow provided, full screening results not yet applicable for protocol | Results; Multimedia Appendix 5 (Preliminary PRISMA-style search flow) |
| DISCUSSION | 19 | Summary of expected contributions | Yes | Discussion |
|  | 20 | Limitations | Yes | Discussion |
|  | 21 | Conclusions | Yes | Discussion |
| FUNDING | 22 | Funding sources | Yes | Funding |
| COI | — | Conflict of interest statement | Yes | COI section |
