# Supplementary material for: Exploring Non-Embodied AI-Based Digital Companions for Older Adults in Aging and Care Contexts: Protocol for a Scoping Review
Source: JMIR Res Protoc. 2026 Jun 24;15:e93196. doi: 10.2196/93196 (PMC13294803; doi:10.2196/93196)
Supplement: Peer Review Report 1 [file resprot-v15-e93196-s006.pdf]

Appendix B: Reviewer Comments and Suggestions

We expect that you and your collaborating researchers will follow the directives provided by applicable institutional, municipal, provincial/territorial, and federal bodies in relation to the COVID-19 pandemic. For example, mitigation strategies may be required to accommodate requirements for physical distancing and limitations on gathering size.

Reviewer 1

Question 1. Does the project significantly contribute to the advancement of knowledge or the development of new applications, and is there an appreciable element of novelty?
The development of conversational AI chatbot in the field of dementia care is already underway with some researchers and tech companies. It is strongly recommended that the research team will try to review what has already been developed or collaborate with these researchers to avoid re-inventing the wheel. The proposed project will be two-years and lots of technological advancement will have changed within the two-year time frame. The research team should plan this project with an open mind that the needs of the technological innovation may change and must be informed by the findings from the end user focus groups.

Question 2. Are the objectives achievable, given the current state of the art and the expertise of the project team?
This research team may benefit from having another researcher with Engineering/Computer Science background who has expertise with AI tech innovations. From the CV, the post-doc trainee doesn't seem to have much experience in tech design and development. Although the supervisor and industry partner have expertise in the area, it is strongly recommended that this team will include another researcher with strong AI background to support the implementation of this project for the duration of the project to enhance feasibility.

Question 3. Are the methods and techniques appropriate for the objectives?
Yes, the methodology of using Collaborative Action Research approaches is appropriate. However, the participatory involvement from people with dementia is not clear. Will they involve mild to moderate and/or advanced stage dementia? Also the intervention is still unclear. It would be helpful to describe what the "AI chatbot" will do and explain how you envision the conversation process and potential features would look like, as well as plan for onboarding/training with the staff/residents to facilitate future adoption. In addition to CFIR outcomes, it would be helpful to clearly define the outcome measures (for people with dementia and caregivers).

Question 4. Is the proposed project at an appropriate level for a postdoctoral fellow?
This project is appropriate to provide training for the post-doctoral fellow in the development and evaluation of technology innovations. The supervisor and academic institution have the appropriate infrastructure and resources to provide support and guidance to the trainee.

Reviewer 2

Question 1. Does the project significantly contribute to the advancement of knowledge or the development of new applications, and is there an appreciable element of novelty?
While there is growing interest (and active research) in studying the role of AI companion for older adults and in general the role of AI in age-care, the proposed project is novel in its potential to generate new knowledge about older adults' acceptance of an AI companion in the context of dementia-care.

Question 2. Are the objectives achievable, given the current state of the art and the expertise of the project team?
The project is appropriately planned.

Question 3. Are the methods and techniques appropriate for the objectives?
The methodology is in general sound, although the approach to building / tailoring the chatbot is not very inclusive -- I am not sure why a Participatory Design approach was not proposed. More importantly, I am concerned about the ethical aspects -- it is not clear if participants will fully understand the privacy implications of using AI technology and what happens to their data (this is not clearly described).

Question 4. Is the proposed project at an appropriate level for a postdoctoral fellow?
The project is suitable for a postdoc although could also be executed by more senior PhD student.

Thanks to our funding partners. Canada Merci à nos bailleurs de fonds.

Alberta British Columbia Research Manitoba New Brunswick Nouveau Newfoundland Labrador Nova Scotia Nouvelle-Écosse Ontario innovationpei Québec Saskatchewan Yukon
